# Supplementary material for: Feeling Connected to Nature Attenuates the Association between Complicated Grief and Mental Health
Source: Int J Environ Res Public Health. 2024 Aug 28;21(9):1138. doi: 10.3390/ijerph21091138 (PMC11431189; doi:10.3390/ijerph21091138)
Supplement: Supplementary file 1 [file ijerph-21-01138-s001.zip › STROBE checklist.pdf]

STROBE Statement—checklist of items that should be included in reports of observational studies

	Item No.	Recommendation	Page No.	Relevant text from manuscript
**Title and abstract**	1	(*a*) Indicate the study's design with a commonly used term in the title or the abstract	1	"To test these hypotheses, we conducted a cross-sectional study." "Cross-sectional analyses indicated that…"
		(*b*) Provide in the abstract an informative and balanced summary of what was done and what was found	1	"We sampled 153 participants…Participants reported CG…Cross-sectional analyses indicated that…"
**Introduction**				
Background/rationale	2	Explain the scientific background and rationale for the investigation being reported	2-4	"Indeed, individuals bereaved because their loved one was infected with COVID-19 tend to experience more symptoms of PGD than individuals grieving deaths by other natural causes…" "Yet, both general grief and complicated grief are comorbid with depression and anxiety." "Meta-analytic results demonstrate that feeling connected to nature is positively associated with overall well-being, psychological well-being…" "However, it has not yet been established whether and how varying degrees of feeling connected to nature…relate to the mental health status of those experiencing complicated grief."
Objectives	3	State specific objectives, including any prespecified hypotheses	4	"To gain a better understanding of the association between grief and mental health, we sought to examine whether nature-related variables (i.e., feeling connected to nature, time spent in nature, residential surrounding greenness) attenuate the association between general grief or complicated grief and indicators of mental health, specifically, depression and anxiety."
**Methods**				
Study design	4	Present key elements of study design early in the paper	4	"We recruited bereaved participants online by posting advertisements…" "After providing informed consent, participants confirmed …Participants then identified the name and passing date…Afterward, we prompted participants to either provide an online link…"

				"Finally, participants completed the survey measures."
Setting	5	Describe the setting, locations, and relevant dates, including periods of recruitment, exposure, follow-up, and data collection	4	"We recruited bereaved participants online by posting advertisements on mTurk, Research Match, and grief group pages on Facebook (i.e., "COVID-19 Loss Support for Family & Friends") and Reddit (i.e., "r/COVIDgrief")." "We recruited participants between 10 March 2021 and 30 January 2022."
Participants	6	(*a*) *Cohort study*—Give the eligibility criteria, and the sources and methods of selection of participants. Describe methods of follow-up *Case-control study*—Give the eligibility criteria, and the sources and methods of case ascertainment and control selection. Give the rationale for the choice of cases and controls *Cross-sectional study*—Give the eligibility criteria, and the sources and methods of selection of participants	4	"We recruited bereaved participants online by posting advertisements on mTurk, Research Match, and grief group pages on Facebook (i.e., "COVID-19 Loss Support for Family & Friends") and Reddit (i.e., "r/COVIDgrief")." "Participants were excluded from the study if they reported that they had not experienced the passing of a close other, were not willing to provide verification of the passing, did not provide verification documents matching the name and date of the person who had passed, were not 18 years or older, or lived outside the United States (see Figure 1)." "Therefore, we excluded data from analyses when participants indicated a term other than eggplant or did not indicate a term as a cautionary measure to remove fraudulent data from the study (see Figure 1)."
		(*b*) *Cohort study*—For matched studies, give matching criteria and number of exposed and unexposed *Case-control study*—For matched studies, give matching criteria and the number of controls per case	N/A	N/A
Variables	7	Clearly define all outcomes, exposures, predictors, potential confounders, and effect modifiers. Give diagnostic criteria, if applicable	5-6	"Participants completed the Brief Grief Questionnaire (BGQ), which is a short screener for complicated grief…higher scores indicating greater complicated grief." "Participants completed a measure of general grief using the Bereavement Experience Questionnaire (BEQ-24)…greater scores indicating greater general grief." "We assessed participants' anxiety using the Generalized Anxiety Disorder Screener (GAD-7)…higher scores indicating greater anxiety." "Participants indicated the extent to which they experienced depressive symptoms in the past week using the PROMIS Short Form v1.0 Depression 8a…Participant's

				standardized t-score served as a measure of depression with higher scores indicating greater depression."<br>"Participants indicated their affective connection to nature based on prior experiences in natural settings using Mayer and Frantz's (2004) Connectedness to Nature scale (CNS)…higher scores indicating feeling more connected to nature."<br>"Participants estimated the amount of time they typically spend in nature…Participants estimated the typical amount of time spent outdoors in nature each day of the week, each day of the month, and each day when the weather permits...higher values indicating more time spent in nature."<br>"We estimated greenness surrounding participants' residential areas using the 5-digit U.S. postal code reported by participants…We calculated Normalized Difference Vegetation Index (NDVI) values of these rectangles to estimate the vegetation level of participants' residential areas…Higher NDVI values thus indicate greater annual average vegetation (i.e., surrounding greenness) in rectangular areas surrounding participants' U.S. postal code regions."
Data sources/ measurement	8*	For each variable of interest, give sources of data and details of methods of assessment (measurement). Describe comparability of assessment methods if there is more than one group	5-6	"Participants completed the Brief Grief Questionnaire (BGQ), which is a short screener for complicated grief…higher scores indicating greater complicated grief."<br>"Participants completed a measure of general grief using the Bereavement Experience Questionnaire (BEQ-24)…greater scores indicating greater general grief."<br>"We assessed participants' anxiety using the Generalized Anxiety Disorder Screener (GAD-7)…higher scores indicating greater anxiety."<br>"Participants indicated the extent to which they experienced depressive symptoms in the past week using the PROMIS Short Form v1.0 Depression 8a…Participant's standardized t-score served as a measure of depression with higher scores indicating greater depression."<br>"Participants indicated their affective connection to nature based on prior experiences in natural settings using Mayer and Frantz's (2004) Connectedness to Nature scale (CNS)…higher scores indicating feeling more connected to nature."<br>"Participants estimated the amount of time they typically spend in nature…Participants estimated the typical amount of time spent outdoors in nature each day of the week, each day of the month, and each day when the weather permits...higher values indicating more time spent in nature."

				"We estimated greenness surrounding participants' residential areas using the 5-digit U.S. postal code reported by participants…We calculated Normalized Difference Vegetation Index (NDVI) values of these rectangles to estimate the vegetation level of participants' residential areas…Higher NDVI values thus indicate greater annual average vegetation (i.e., surrounding greenness) in rectangular areas surrounding participants' U.S. postal code regions."
Bias	9	Describe any efforts to address potential sources of bias	4-5, 7	"We sampled from diverse online data collection platforms to minimize sampling bias." "We intended to identify fraudulent online participants (i.e., participants concealing a device location outside the United States using virtual private servers to conceal internet provider addresses)…" "Therefore, we used responses to the BGQ as a continuous variable. We summed items to create a measure of complicated grief (α = .81) with higher scores indicating greater complicated grief." "We did not substitute or impute data in cases of missing data."
Study size	10	Explain how the study size was arrived at	7	"We recruited as many participants as possible from various platforms over a 10-month period while the survey was available. The sample size, thus, reflects the number of individuals we were able to access who had experienced COVID-19 bereavement and provided sufficient data for us to deem their responses as valid.
Quantitative variables	11	Explain how quantitative variables were handled in the analyses. If applicable, describe which groupings were chosen and why	5-7	"We summed items to create a measure of complicated grief (α = .81) with higher scores indicating greater complicated grief." "Instead, we averaged items into a measure of general grief (α = .94) with greater scores indicating greater general grief." "We summed items to create a measure of anxiety (α = .95) with higher scores indicating greater anxiety." "We summed items into a raw score which we then transformed into a standardized t-score (M=50, SD=10) for each participant using the appropriate score conversion chart. Participant's standardized t-score served as a measure of depression (α = .96) with higher scores indicating greater depression." "We averaged items to create a measure of feeling connected to nature (α = .80) with higher scores indicating feeling more connected to nature."

				"We summed items to create a time spent in nature measure (α = .90) with higher values indicating more time spent in nature."<br>"First, we identified the geographical spaces associated with postal code areas, which were irregularly shaped. Then, we fit rectangles around these geographical spaces. Next, we used the latitudinal and longitudinal coordinates of the centermost point as well as the length and width of these rectangles to define the geographical areas nearest to where participants lived. Finally, we calculated Normalized Difference Vegetation Index (NDVI) values of these rectangles to estimate the vegetation level of participants' residential areas… We extracted NDVI values for each postal code area using the MODIS Global Subsets Tool (https://modis.ornl.gov/globalsubset), similar to previous research on postal code vegetation…We averaged mean NDVI values in the postal code area for each image taken between the date the participant took the survey and a year prior to the participation date. If an image was not taken exactly on the date a year prior to a survey response, we used the mean NDVI value from the image that was taken at the next date. Higher NDVI values thus indicate greater annual average vegetation (i.e., surrounding greenness) in rectangular areas surrounding participants' U.S. postal code regions."
Statistical methods	12	(*a*) Describe all statistical methods, including those used to control for confounding	7	"First, we calculated Pearson's correlations between study variables. Next, we conducted simple moderation analyses to test study hypotheses…We further probed any significant ($p<.05$) or marginally significant ($p<.10$) simple moderation by conducting simple slope analyses, examining the effect of complicated grief or general grief on depression or anxiety at varying levels (i.e., 1 standard deviation below the mean, the mean, and 1 standard deviation above the mean) of nature-related variables."
		(*b*) Describe any methods used to examine subgroups and interactions	7	"We further probed any significant ($p<.05$) or marginally significant ($p<.10$) simple moderation by conducting simple slope analyses, examining the effect of complicated grief or general grief on depression or anxiety at varying levels (i.e., 1 standard deviation below the mean, the mean, and 1 standard deviation above the mean) of nature-related variables."
		(*c*) Explain how missing data were addressed	7	"Nevertheless, we retained these participants when they had provided sufficient data (i.e., answered most questions for a measure) for a particular set of analyses. We did not substitute or impute data in cases of missing data."

		(*d*) *Cohort study*—If applicable, explain how loss to follow-up was addressed<br>*Case-control study*—If applicable, explain how matching of cases and controls was addressed<br>*Cross-sectional study*—If applicable, describe analytical methods taking account of sampling strategy	N/A	N/A
		(*e*) Describe any sensitivity analyses	N/A	N/A
**Results**				
Participants	13*	(a) Report numbers of individuals at each stage of study—eg numbers potentially eligible, examined for eligibility, confirmed eligible, included in the study, completing follow-up, and analysed	5	"Flow diagram of participant exclusion."
		(b) Give reasons for non-participation at each stage	4-5, 7	"We deemed responses valid if….Participants were excluded from the study if they reported that they had not experienced the passing of a close other…"<br>"Flow diagram of participant exclusion."<br>"Not all participants completed all measures in full…"
		(c) Consider use of a flow diagram	5	"Flow diagram of participant exclusion."
Descriptive data	14*	(a) Give characteristics of study participants (eg demographic, clinical, social) and information on exposures and potential confounders	7	"Participants included 153 bereaved individuals (109 females, *M*age=42.1…"<br>"Though we recognize the current sample may not be perfectly comparable to other samples, descriptive statistics of reported general grief (*M*=1.91, *SD*=0.69)…."
		(b) Indicate number of participants with missing data for each variable of interest	7	"Not all participants completed all measures in full. In some cases, participants did not provide any response to measures of anxiety (*n*=1)…"
		(c) *Cohort study*—Summarise follow-up time (eg, average and total amount)	N/A	N/A
Outcome data	15*	*Cohort study*—Report numbers of outcome events or summary measures over time	N/A	N/A

		Case-control study—Report numbers in each exposure category, or summary measures of exposure	N/A	N/A
		Cross-sectional study—Report numbers of outcome events or summary measures	7-8	"Finally, participants reported elevated levels of anxiety (M=7.40, SD=6.41) and depression (M=55.51, SD=10.78). Though the mean anxiety level in the sample reflects above-average levels of anxiety, mean anxiety scores in the sample reflect mild levels of anxiety remaining within non-clinical ranges. Similarly, mean depression levels in the sample reflect mild to moderate levels of depression, nearing a value (i.e., 60) indicating clinical significance."
Main results	16	(*a*) Give unadjusted estimates and, if applicable, confounder-adjusted estimates and their precision (eg, 95% confidence interval). Make clear which confounders were adjusted for and why they were included	8-10	"Indeed, feeling connected to nature attenuated the association between complicated grief and depression, $b = -1.36$, $SE = .58$, $t(148) = -2.34$, $p = .021$, 95% CI [-2.50, -0.21]."<br>"Specifically, feeling connected to nature attenuated the association between complicated grief and anxiety, though this moderation only approached conventional levels of significance, b = -.61, SE = .34, t(148) = -1.78, p = .077, 95% CI [-1.29, 0.07]."<br>"In contrast, feeling connected to nature did not moderate the associations of general grief with depression or anxiety, bs ≤ -.85, SEs ≥ 1.35, ts(148) ≤ -.47, ps ≥ .47, though we found that general grief predicted depression and anxiety, bs ≤ 10.30, SEs ≤ .97, ts(150) ≤ 10.64, ps < .001. Neither time spent in nature nor surrounding greenness moderated the associations of general or complicated grief with depression or anxiety, bs ≤ 7.06, SEs ≥ .04, ts ≤ 1.22, ps ≥ .22."
		(*b*) Report category boundaries when continuous variables were categorized	N/A	N/A
		(*c*) If relevant, consider translating estimates of relative risk into absolute risk for a meaningful time period	N/A	N/A
Other analyses	17	Report other analyses done—eg analyses of subgroups and interactions, and sensitivity analyses	8-9	"We probed this moderation by conducting simple slope analyses of the association between complicated grief and depression at different levels of feeling connected to nature. There was a significant association between complicated grief and depression at low levels of feeling connected to nature (b = 2.23, SE = .44, t(148) = 5.01, p < .001, 95% CI [1.35, 3.11]) and medium levels of feeling connected to nature (b = 1.49, SE

				= .30, t(148) = 5.05, p < .001, 95% CI [0.91, 2.07]). In contrast, at high levels of feeling connected to nature, the association between complicated grief and depression was non-significant (b = .75, SE = .42, t(148) = 1.78, p = .08, 95% CI [-0.08, 1.58]).”<br>“Similar to the pattern observed when testing the association between complicated grief and depression, simple slope analyses revealed a significant association between complicated grief and anxiety at low levels of feeling connected to nature (b = 1.30, SE = .26, t(148) = 4.94, p < .001, 95% CI [0.78, 1.82]) and medium levels of feeling connected to nature (b = .96, SE = .17, t(148) = 5.54, p < .001, 95% CI [0.62, 1.31]). At high levels of feeling connected to nature, the association between complicated grief and anxiety was weaker in size but remained significant (b = .63, SE = .25, t(148) = 2.54, p = .012, 95% CI [0.14, 1.12]).”
**Discussion**				
Key results	18	Summarise key results with reference to study objectives	10	“Given previous evidence that feeling connected to nature, more time spent in nature, and more residential greenness are associated with better mental health, we tested whether these variables moderated the association between grief and poor mental health following experiencing the loss of a close other by COVID-19 infection. Specifically, we provide evidence that feeling connected to nature attenuates the association between complicated grief and depression…”
Limitations	19	Discuss limitations of the study, taking into account sources of potential bias or imprecision. Discuss both direction and magnitude of any potential bias	11-12	“Our study also has limitations. First, causal interpretation of our data is limited due to the data’s correlational nature.”<br>“Our reliance on correlational data also limits our understanding of the mechanisms by which feeling connected to nature attenuates the association between complicated grief and reduced mental health.”<br>“Another limitation of our study is having a non-representative sample.”<br>“Moreover, though we required participants submit verifying documents to corroborate self-reported loss, this study is limited by not requiring participants to corroborate self-reports of close-other passing with a death certificate.”<br>“Another limitation of the current study is that important study variables are based on participants’ self-report rather than direct observations of participants’ thoughts and behaviors.”

				“Another potential limitation of the current study is the resolution at which surrounding greenness images were taken.” “Finally, our study may be limited in power, despite attempts to recruit an adequate sample of grieving persons.”
Interpretation	20	Give a cautious overall interpretation of results considering objectives, limitations, multiplicity of analyses, results from similar studies, and other relevant evidence	10	“We thus conclude that feeling connected to nature—not simply spending more time in nature or being surrounded by nature—may serve an important role in the mental health status of people experiencing complicated grief.”
Generalisability	21	Discuss the generalisability (external validity) of the study results	12	“Specifically, our sample was largely made up of females and White- or European American-identifying individuals, limiting the generalizability of study conclusions to individuals outside of these demographics.”
**Other information**				
Funding	22	Give the source of funding and the role of the funders for the present study and, if applicable, for the original study on which the present article is based	13	“This research was funded by the start-up funds of Dr. Dominik Mischkowski at Ohio University. No additional external funding was used.”

*Give information separately for cases and controls in case-control studies and, if applicable, for exposed and unexposed groups in cohort and cross-sectional studies.

Note: An Explanation and Elaboration article discusses each checklist item and gives methodological background and published examples of transparent reporting. The STROBE checklist is best used in conjunction with this article (freely available on the Web sites of PLoS Medicine at http://www.plosmedicine.org/, Annals of Internal Medicine at http://www.annals.org/, and Epidemiology at http://www.epidem.com/). Information on the STROBE Initiative is available at www.strobe-statement.org.
